# Machine-Learning Prediction of Postoperative Pituitary Hormonal Outcomes in Nonfunctioning Pituitary Adenomas: A Multicenter Study

**DOI:** 10.3389/fendo.2021.748725

**Published:** 2021-10-07

**Authors:** Yi Fang, He Wang, Ming Feng, Wentai Zhang, Lei Cao, Chenyu Ding, Hongjie Chen, Liangfeng Wei, Shuwen Mu, Zhijie Pei, Jun Li, Heng Zhang, Renzhi Wang, Shousen Wang

**Affiliations:** ^1^ Department of Neurosurgery, The Fuzong Clinical Medical College of Fujian Medical University, Fuzhou, China; ^2^ Department of Neurosurgery, The Fuzhou General Hospital, Fuzhou, China; ^3^ Department of Neurosurgery, The Peking Union Medical College Hospital, Chinese Academy of Medical Sciences and Peking Union Medical College, Beijing, China; ^4^ Department of Neurosurgery, The Tiantan Hospital, Capital Medical University, Beijing, China; ^5^ Department of Neurosurgery, The First Affiliated Hospital of Fujian Medical University, Fuzhou, China

**Keywords:** hypopituitarism, machine learning, neuroendocrine tumor, pituitary tumors, prognosis, surgery

## Abstract

**Objective:**

No accurate predictive models were identified for hormonal prognosis in non-functioning pituitary adenoma (NFPA). This study aimed to develop machine learning (ML) models to facilitate the prognostic assessment of pituitary hormonal outcomes after surgery.

**Methods:**

A total of 215 male patients with NFPA, who underwent surgery in four medical centers from 2015 to 2021, were retrospectively reviewed. The data were pooled after heterogeneity assessment, and they were randomly divided into training and testing sets (172:43). Six ML models and logistic regression models were developed using six anterior pituitary hormones.

**Results:**

Only thyroid-stimulating hormone (*p* < 0.001), follicle-stimulating hormone (*p* < 0.001), and prolactin (PRL; *p* < 0.001) decreased significantly following surgery, whereas growth hormone (GH) (*p* < 0.001) increased significantly. The postoperative GH (*p* = 0.07) levels were slightly higher in patients with gross total resection, but the PRL (*p* = 0.03) level was significantly lower than that in patients with subtotal resection. The optimal model achieved area-under-the-receiver-operating-characteristic-curve values of 0.82, 0.74, and 0.85 in predicting hormonal hypofunction, new deficiency, and hormonal recovery following surgery, respectively. According to feature importance analyses, the preoperative levels of the same type and other hormones were all important in predicting postoperative individual hormonal hypofunction.

**Conclusion:**

Fluctuation in anterior pituitary hormones varies with increases and decreases because of transsphenoidal surgery. The ML models could accurately predict postoperative pituitary outcomes based on preoperative anterior pituitary hormones in NFPA.

## Introduction

Hypopituitarism is a deficiency in one or several pituitary hormones (1). The pituitary gland is subdivided into adenohypophysis (anterior pituitary) and neurohypophysis (posterior pituitary). Adenohypophysis primarily produces six hormones: growth hormone (GH), thyroid-stimulating hormone (TSH), follicle-stimulating hormone (FSH), luteinizing hormone (LH), prolactin (PRL), and adrenocorticotropic hormone (ACTH). Neurohypophysis is responsible for storing and releasing oxytocin and antidiuretic hormones derived from the hypothalamus. Sellar lesions, including pituitary adenoma (PA), are commonly associated with deficiencies in pituitary hormones because of compression of adenohypophysis and pituitary stalk vessels (1). Nonfunctioning pituitary adenoma (NFPA) is a common factor in hypopituitarism, with a prevalence ranging from 37% to 85% (2–4). NFPAs include gonadotroph adenomas, silent PAs, and null cell adenomas, and they are characterized by mass effects, as detailed in the last WHO classification (4, 5). Transsphenoidal surgery is the primary treatment for NFPAs. Although surgery could treat NFPA-induced hypopituitarism, secondary hypopituitarism is a common consequence of pituitary surgery (6). The incidence of surgery-induced hypopituitarism varies from 2% to 15% (2, 3, 7).

Adenohypophysis hypofunction could cause severe hormonal disorders, which are responsible for decreasing the quality of life in patients. Two recent meta-analyses have revealed excess mortality in patients suffering from hypopituitarism (8, 9). Perioperative management of hormones is an essential part of treatment for patients undergoing pituitary surgery. Therefore, anticipating and monitoring hypopituitarism and adjusting therapeutic strategies are necessary to minimize the effect of hypopituitarism on prognosis.

Multicenter studies of perioperative anterior pituitary hormones in NFPAs are limited. In the present study, the clinical characteristics of NFPAs in four medical centers were retrospectively reviewed. The interrelations between preoperative anterior pituitary hormones and postoperative secretion of adenohypophysis were analyzed. Machine learning (ML) models were developed to predict the secretory capacity of the anterior pituitary. Feature importance analysis was also performed for model explanation. This study aimed to investigate the perioperative hormonal characteristics of the anterior pituitary gland and provide a framework for ML models to predict postoperative hormone deficiency in the adenohypophysis.

## Materials And Methods

A collected database (CPDRN) was used as a screening tool in the cohort. Clinical data were obtained from Fuzhou General Hospital (FGH), Peking Union Medical College Hospital (PUMCH), the First Affiliated Hospital of Fujian Medical University (FAHFMU), and Beijing Tiantan Hospital (BTH). This study was approved by the Institutional Review Boards of all the medical centers. As it was a retrospective study, the patients did not sign an informed consent.

### Data Preparation

In the cohort, a total of 215 male adult patients who underwent NPFA resection between 2015 and 2021 were reviewed retrospectively (FGH, n = 111; PUMCH, n = 41; FAHFMU, n = 20; and BTH, n = 43). The age ranged from 18 years to 79 years.

The criteria for admission were as follows:

1. Macroadenomas (more than 1 cm) with hormonal and histopathological diagnosis of NFPA.
2. Male adult patients undergoing perioperative neuroendocrine evaluation of six anterior pituitary hormones.
3. Patients without previous radiotherapy and surgery of pituitary diseases.
4. Patients without a history of long-term pharmacotherapy and endocrine diseases that could affect adenohypophysis function.

### Evaluation of the Secretory Capacity of Each Anterior Pituitary Hormone

All six anterior pituitary hormones (GH, TSH, FSH, LH, PRL, and ACTH) were monitored based on the serum hormone levels to reflect the secretory capacity of the adenohypophysis. The patients received a neuroendocrine evaluation and re-evaluation within 7 days before and after surgery. However, the random GH level lower limit for adults was 0 μg/L, and confirmatory stimulation tests were not routinely conducted to diagnose GH deficiency in all centers. Thus, the deficiencies of the remaining five hormones were evaluated. Pituitary hormonal deficiency (PHD) was defined as one or more inappropriately low anterior pituitary hormones. New deficiency was defined as at least one normal hormone secreted before the present postoperative deficiency. Recovered deficiency was defined as at least one of the preoperative deficits that returned to normal function following surgery (6).

Although details of the hormonal tests were not standardized, the hormonal reference ranges of the four medical centers were similar, following the conversion of the unit. Furthermore, the heterogeneity across hormonal findings was limited (**Supplementary Material Figures 1** and **2**). Consequently, the multicenter hormonal outcomes were pooled to facilitate the training of the predictive models. The principal investigator from each center assessed the hormonal outcomes separately. The adjudicated results were forwarded to the coordinating site for further analysis.

### Model Training and Explanation

Six common supervised ML models were developed to predict the postoperative secretory capacity of the adenohypophysis: decision tree (DT), random forest (RF), K-nearest neighbor (KNN), AdaBoost algorithm, support vector machine (SVM), and neural network (NN). In addition, the logistic regression (LR) model was trained as the reference frame for comparison with ML models. In the cohort, 215 male patients with NFPA underwent a fivefold division (172:43) of the randomized dataset. These dichotomous models for postoperative endocrine evaluation were trained using only the six anterior pituitary hormones representing the secretory capacity of the adenohypophysis.

Hyperparameters are parameters that are not directly learned within estimators. They are passed as arguments to the constructor of the estimator classes. GridSearchCV provided by the Scikit-Learn library enabled efficient parameter search strategies. It could search for and evaluate all possible combinations from a grid of parameter values (10). Parameter search used the area under the receiver operating characteristic curve (AUC-ROC) of estimators to evaluate the parameter setting, following the convention that higher return values are better than lower return values. Finally, the parameter combination that outputs the maximum AUC-ROC value was obtained. The parameters were further optimized by manually performing fine-tuning parameter correction.

The abovementioned models were trained and tested using fivefold cross-validation. An evaluation was performed on one group by using the model built on 80% of the cases. Sensitivity, specificity, and AUC-ROC were provided. The algorithm with the highest AUC-ROC was considered as the optimal model.

The evaluation of feature importance on ML classification models facilitated the improvement of model interpretability.

### Statistical Analyses and Software Application

Stata (version 19.0) was used to analyze the heterogeneity from different medical centers with the fixed-effect model. Q test (*p* < 0.10, indicating heterogeneity) and I^2^ test (I^2^ > 50%, indicating moderate-to-high heterogeneity) were performed. Jupyter Notebook, an open-source project, served as a code execution tool (compatible with python and R environments) (11, 12). Python (version 3.9) was run for model building and training. Various open-source ML libraries, including Scikit-Learn (version 0.24.2), were used (13). All data analyses and data visualization were processed in R (version 4.0.4) and python (version 3.9). Categorical variables were analyzed using the chi-square test. An independent sample t-test was used to compare the differences in hormonal levels. A two-sided *p*-value of less than 0.05 was considered significant.

## Results

### Clinical Characteristics

A total of 215 male patients with NFPA from the four medical centers were included (**Table 1**). In the cohort, 94/215 (43.7%) patients had a preoperative deficiency in at least one of the evaluated hormones. The incidence of preoperative deficiencies in individual hormonal axes was 72 (33.5%) for LH, 19 (8.8%) for TSH, 14 (6.5%) for ACTH, 13 (6.0%) for PRL, and 10 (4.7%) for FSH. The frequency of deficits in postoperative hormones (n = 139/215, 64.7%) was significantly higher than that in preoperative hormones (*p* < 0.001). The incidence of postoperative deficiencies of individual pituitary hormone was 95/139 (68.3%) for LH, 50/139 (36.0%) for TSH, 41 (29.5%) for PRL, 17 (12.2%) for FSH, and 13 (9.4%) for ACTH. A new deficiency in at least one hormone following surgery was observed in 41.9% (90/215) of patients, whereas 53/215 (24.7%) cases had normal hormonal levels prior to surgery. A new deficiency was most likely to be observed in TSH (17.3%) and LH levels (19.6%) and less likely to be observed in ACTH levels (4.5%). Of the patients with a preoperative deficiency, 21/94 (22.3%) recovered in one or more deficient hormones. However, 13/21 (61.9) patients still had postoperative hormone deficits, and a new deficiency was observed in 9/21 (42.9%) cases.

**Table 1:** Summary of characteristics.

Characteristics	No./Total
**No. of patients**	215
**Age in yrs**	51.8 ± 11.8
**Tumor diameter**	29.3 ± 12.8
**Preoperative PHD**	94/215 (43.7)
TSH	19/215 (8.8)
FSH	10/215 (4.7)
LH	72/215 (33.5)
PRL	13/215 (6.0)
ACTH	14/215 (6.5)
Deficiency in 1 hormone axis	58/94 (61.8)
Deficiency in 2 hormone axes	18/94 (19.1)
Deficiency in ≥ 3 hormone axes	18/94 (19.1)
**Resection goal**	
Gross total resection	151/200 (75.5)
Subtotal resection	49/200 (24.5)

Further details of the perioperative results are shown in **Tables 2** and **3**. The postoperative GH levels were significantly higher than the preoperative levels (*p* < 0.001), whereas the TSH (*p* < 0.001), FSH (*p* < 0.001), and PRL (*p* < 0.001) levels significantly decreased after surgery. Perioperative hormonal level fluctuations were not significant for LH (*p* = 0.96) and ACTH (*p* = 0.77). All patients underwent transsphenoidal surgical resection. A total of 151/200 (75.5) patients underwent gross total resection (GTR). Subtotal resection (STR) was observed in 24.5% (49/200) of patients. The GH (*p* = 0.07) levels were slightly higher in the GTR group than in the STR group after surgery. However, the PRL secretion levels were significantly lower in patients with GTR (*p* = 0.03). Postoperative LH (*p* = 0.25) and FSH (*p* = 0.41) presented an increase in tendency following GTR, with no significant differences.

**Table 2:** Anterior pituitary hormone secretion after transsphenoidal surgery for NFPAs.

Parameter	No. (%)
**Postoperative hormonal hypofunction**	139/215 (64.7)
Deficiency in 1 hormone axis	84/139 (60.4)
Deficiency in 2 hormone axes	41/139 (29.5)
Deficiency in ≥ 3 hormone axes	14/139 (10.1)
**Individual hormonal function**	
TSH deficiency	50
New deficiency	34/196 (17.3)
Deficiency resolved	3/19 (15.8)
FSH deficiency`	17
New deficiency	9/205 (4.4)
Deficiency resolved	2/10 (20.0)
LH deficiency	95
New deficiency	28/143 (19.6)
Deficiency resolved	5/72 (6.9)
PRL deficiency	41
New deficiency	29/201 (14.4)
Deficiency resolved	1/13 (7.7)
ACTH deficiency	13
New deficiency	9/202 (4.5)
Deficiency resolved	10/14 (71.4)
**Any new deficiency in ≥1 hormone axes**	90/215 (41.9)
**Any improvement in ≥1 hormone axes**	21/94 (22.3)

**Table 3:** Comparison of anterior pituitary hormone levels.

Hormonecategories	Perioperative fluctuation			Tumor resection^a^		
	Preoperative Mean ± SD(n=215)	Postoperative Mean ± SD(n=215)	*p*-value	STR Mean ± SD(n=49)	GTR Mean ± SD(n=151)	*p*-value
**GH**	0.24 ± 0.29	0.69 ± 1.00	<**0.001↑**	0.48 ± 0.46	0.78 ± 1.14	0.07**↑**
**TSH**	1.75 ± 1.24	0.96 ± 0.90	<**0.001↓**	1.01 ± 0.94	0.93 ± 0.83	0.58
**FSH**	6.49 ± 5.80	4.94 ± 3.84	**0.001↓**	4.69 ± 4.09	5.20 ± 3.72	0.41
**LH**	2.66 ± 2.22	2.61 ± 2.42	0.96	2.29 ± 3.45	2.82 ± 2.57	0.25
**PRL**	11.90 ± 7.12	5.19 ± 3.87	<**0.001↓**	6.27 ± 4.65	4.94 ± 3.44	**0.03↓**
**ACTH**	21.86 ± 14.04	21.11 ± 18.33	0.77	21.97 ± 17.68	20.80 ± 21.64	0.73

a Postoperative hormone levels.

GTR, gross total resection; SD, standard deviation; STR, subtotal resection.

The bold values represent statistical significance (p < 0.05).

### Predictive Models for Postoperative Hypofunction

**Figure 1:**
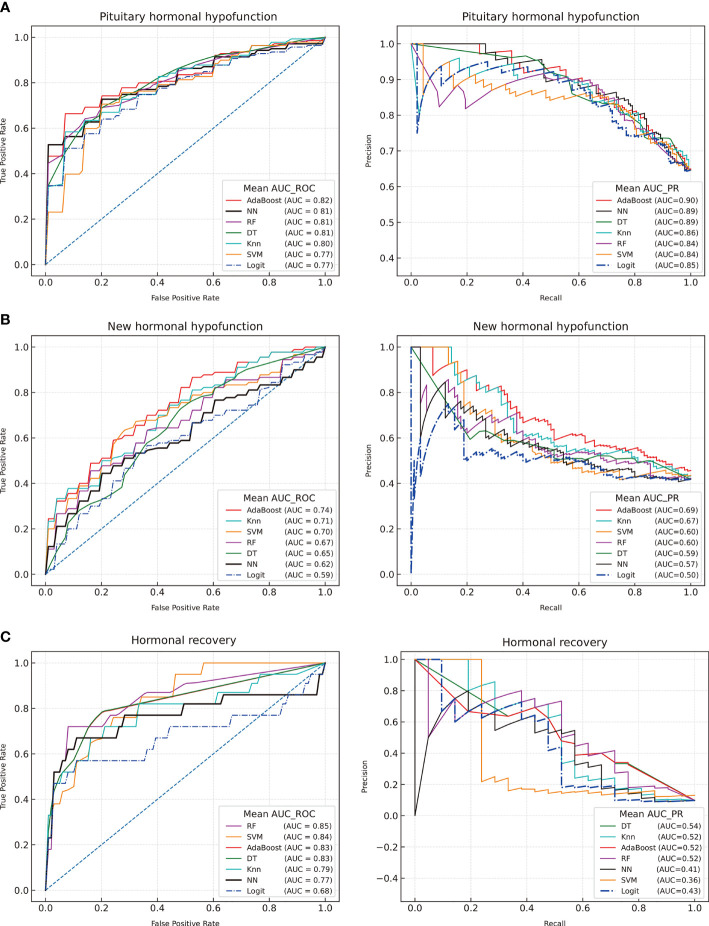
Receiver operating characteristic curves and precision-recall curves of machine learning for the postoperative outcomes of the adenohypophysis. **(A)** Postoperative hormonal hypofunction; **(B)** New hormonal hypofunction; **(C)** Hormonal recovery. Decision tree (DT), Random Forest (RF), K-nearest neighbor (KNN), Adaptive Boosting algorithm (AdaBoost), Support Vector Machine (SVM), Neural Network (NN), logistic regression (Logit).

The six preoperative anterior pituitary hormones were considered as input features for the dichotomous models of postoperative hypopituitarism. The predictive capabilities were compared after training and fivefold cross-validation of the eight models. The performance details of each model are shown in **Figure 1**.

The AdaBoost model was considered as the optimal model based on the AUC-ROC results (AUC = 0.82, AUC-PR = 90), followed by NN (AUC-ROC = 0.81, AUC-PR = 0.89), RF (AUC-ROC = 0.81, AUC-PR = 84), DT (AUC-ROC = 0.81, AUC-PR = 0.89), KNN (AUC-ROC = 0.80, AUC-PR = 0.86), and SVM (AUC-ROC = 0.77, AUC-PR = 0.84). Based on the AUC value of the ROC and PR curve, the ML models were slightly better than the LR model (AUC-ROC = 0.78, AUC-PR = 0.86).

### Predictive Models for New Postoperative Hypofunction and Hormonal Recovery

The ML models were selected to predict new postoperative hypofunction and hormonal remission. GridSearchCV was reset for predictive models. The optimal model for the prediction of surgery-induced PHD revealed an AUC-ROC of 0.74 and an AUC-PR of 0.69. Hormonal recovery was also assessed using these models. The optimal model for postoperative recovery prediction had an AUC-ROC of 0.85 and an AUC-PR of 0.54. The LR models achieved AUC-ROC values of 0.59 and 0.68 in predicting new postoperative hypofunction and hormonal recovery, respectively. The ML models presented a higher reliable performance in predicting hormonal outcomes than the LR model.

### Predictive Models for Individual Postoperative Hypopituitarism and Explanation

Predictive models for secondary PHD following surgery were reported in previous studies. However, these models could not support more informative details for individual hormonal outcomes. Thus, predictive models for postoperative uniaxial PHD were established and adjusted on the basis of the parameters recommended by GridSearchCV.

**Figure 2:**
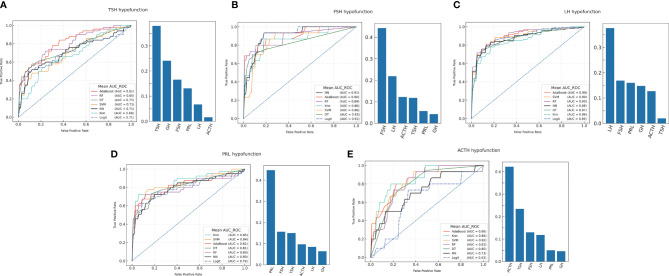
The machine learning models prediction of individual hormonal deficiency and feature importance analysis. **(A)** TSH hypofunction; **(B)** FSH hypofunction; **(C)** LH hypofunction; **(D)** PRL hypofunction; **(E)** ACTH hypofunction.

The ML models were constructed to analyze and compare the performance of preoperative hormones in predicting postoperative individual hypopituitarism (**Figure 2** and **Supplementary Figure 3**). The results of model construction suggested that ML models could be adjusted to accurately predict different PHD compared with the LR model.

The feature importance of anterior pituitary hormones on the optimal model was evaluated to explain the predictive models. Feature importance indicated that the individual hormonal deficiency was related to the same hormonal axis and the other anterior pituitary hormones prior to surgery (**Figure 2**). Postoperative anterior pituitary hormonal deficiency was associated with multiple hormone secretion.

## Discussion

In previous studies, patients with large-volume, suprasellar-extension, and invasive PAs likely have postoperative pituitary hypofunction (1, 6, 14). Surgery is the first-line treatment to decompress NFPA, but it is also regarded as a trigger to hormonal hypofunction because of intraoperative traction and normal pituitary tissue trauma (3, 7, 15, 16). These extrinsic factors affect the anterior pituitary hormone secretion and induce hormonal fluctuation. However, few studies directly assessed the outcomes of the adenohypophysis in accordance with the change characteristics of the perioperative hormonal level in NFPAs. In these studies, male and female hormones were generally pooled for assessment (3, 17, 18). Significant differences in hormonal levels were found between men and women. Furthermore, the female hormonal levels presented significant differences in different physiological periods. The evaluation of the menstrual period in retrospective studies was dependent on patient-reported personal history, thereby lacking reliable and objective evidence. Errors might be found in the assessment of female hormones. High-quality data are necessary for an accurate and reliable clinical model. Consequently, only male patients with NFPA from four medical centers were included in this study to reflect the perioperative fluctuation of anterior pituitary hormone accurately and objectively.

### Effects of Surgery on Hormonal Levels

Although transsphenoidal surgery could be invariably recommended for the decompression of NFPA on the pituitary gland, surgery could not treat tumor-induced hypopituitarism immediately. On the contrary, transsphenoidal surgery had a significant risk of sacrificing the remaining adenohypophysis function (14). Andrew et al. reported that 21.1% of NFPA cases experienced recovery at the 6-month follow-up. Kim et al. reported 15.4% gland improvement in patients with NFPA. Although hormonal improvement in at least one hormonal axis was observed in 22.3% of patients in the current study, nine out of the 21 cases suffered from a new deficiency in other hormonal axes in the immediate postoperative period.

Interestingly, not all hormonal axes decreased following surgery. Increased postoperative hormones could be observed in the GH. The mass effects of the pituitary tumor might have fewer effects on pituitary somatotroph secretion than on other hormones. However, the mechanism still remains uncertain. Increased postoperative GH was also reported in a cohort with 164 NFPAs (3). Kobayashi et al. evaluated GH secretion in response to GH-releasing peptide-2 (GHRP2) and found 94/119 NFPAs diagnosed with GH deficiency after surgery (3). The limitation of this study is that GHRP2 was performed at 2 weeks and 1–2 years after surgery, and the short-term GH dynamics were not analyzed. Increased GH hormone might be a reaction against surgical stress in major surgery, which could return to normal within seven days (19, 20).

The effects of resection goals on perioperative hormones were also evaluated in this study. The postoperative GH levels in GTR were slightly higher, but the PRL levels were significantly lower than those in STR. GTR presents a higher degree of intraoperative destruction than STR. Therefore, the GH levels in GTR cases are higher than those in STR against surgical stress. Increased PRL in NFPAs frequently occurs because of inhibitory dopamine transportation from the hypophyseal portal system (21, 22). GTR could effectively relieve the compression of the pituitary stalk than STR. Thus, the postoperative PRL levels in GTR were slightly lower than those in STR.

### ML Predictive Models for Postoperative Hypopituitarism

The causes of fluctuation differences in anterior pituitary hormones remain uncertain. Hypertension, tumor diameter, invasion, surgical trauma, and residual tumor were considered predictors of pituitary hormonal prognosis. In clinical practice, partial hormonal deficiencies frequently occur in patients with NFPA compared with panhypopituitarism. In the current study, 80.9% and 89.9% of patients with hormonal deficits had hypofunction in one or two hormonal axes before and after surgery, respectively. Except for PRL, other anterior pituitary hormones could also be higher than the normal reference ranges in NFPAs. These extrinsic factors could not well explain the complicated hormonal fluctuation and support more detailed information. Hormonal levels could show the dynamic response of adenohypophysis function during the perioperative period in detail. Therefore, the postoperative hormonal results from preoperative anterior pituitary hormones were predicted and explained using ML models. ML is a modern predictive statistical method widely used to conduct prediction models in neurological studies, such as surgical remission of acromegaly, GTR of PAs, and cerebral spinal fluid leakage. The researchers were the first to train and explain the ML models of postoperative hypofunction of adenohypophysis and predict the postoperative outcomes of anterior pituitary secretion. In addition, the ML models were proven reliable to predict hormonal outcomes in NFPAs than the LR model.

In this multicenter study, measures to improve data quality were used for stable and reliable ML models, including only male patients to avoid hormonal heterogeneity from different sexes, heterogeneity analysis and combining data from the four centers, and cross-validation. Furthermore, prediction models were constructed by only using the six hormones that adenohypophysis directly secretes, and they did not include extrinsic factors (tumor diameter, invasion, and suprasellar extension). After these noise variables were minimized, the preoperative secretory capacity of adenohypophysis could effectively predict the prognosis following transsphenoidal surgery. The optimal predictive model for postoperative hormonal deficits, new hormonal hypofunction, and hormonal recovery had AUC-ROC values of 0.82, 0.74, and 0.86, respectively, which were better than those in conventional statistics, such as the LR model.

Individual hormonal hypofunction was also analyzed and explained using ML models. In the prediction of individual hormonal deficits, the optimal ML models achieved AUC-ROC ranging from 0.81 to 0.91. The ML models could aid in predicting the secretory capacity of adenohypophysis and the prognosis of individual hormonal axes. The results of feature importance analysis implied that multiple relations were found among various anterior pituitary hormones. Individual hormonal levels are the main factor for the prediction of postoperative outcomes. Moreover, the other hormones of the anterior pituitary are considered as important factors for individual hormonal outcomes. The assessment of the whole pituitary hormones could accurately predict the remnant pituitary function after surgery (17).

Several limitations warrant further discussion. In the multicenter study, details of the hormonal tests were not standardized, including the testing reagents and testing methods. Although we pooled multicenter hormonal outcomes after heterogeneity assessment, bias in measurements at different centers remained. GH deficiency was not available in the cohort. If available, then it could increase the prediction accuracy. Peripheral hormones were not analyzed because this study focused on the secretory capacity of the adenohypophysis. In addition, this study could only reflect the characteristics of hormonal changes during the perioperative period. The long-term prognosis of adenohypophysis requires further study design for analysis. The current number of cases also remains limited. Building an accurate and reliable model require high-throughput data collection and inclusion of female patients with an accurate physiological period record. These limitations were attributed to the deficiencies of retrospective studies.

## Conclusions

The characteristics of individual anterior pituitary hormone fluctuation were not consistent in the short term after NFPA resection. In addition to mass effects and surgical trauma, preoperative hormonal levels must be the focus of concern. Perioperative evaluation through hormonal levels could reflect and predict the secretory function of the adenohypophysis in NFPA. The ML model is a subtype of artificial intelligence, which could be integrated into clinical application as an NFPA prognosis prediction. The reliability and practicability of the prediction were also confirmed in this study. The combination of multiple models facilitated the selection of accurate predictive models and increased interpretability. The ML models could be an essential technical tool for future clinical prediction.

## Data Availability Statement

The raw data supporting the conclusions of this article will be made available by the authors, without undue reservation.

## Ethics Statement

The studies involving human participants were reviewed and approved by the Institutional Review Board of Fuzhou General Hospital of Fujian Medical University and Peking Union Medical College Hospital. The patients/participants provided their written informed consent to participate in this study.

## Author Contributions

YF, HW, and MF contributed equally to the present study. All authors contributed to the article and approved the submitted version.

## Funding

The present study was funded by the Fujian Provincial Key Project of Science and Technology Plan (grant no. 2018Y0067) and the Fujian Medical University Sailing Fund Project (grant no. 2019QH2043).

## Conflict of Interest

The authors declare that the research was conducted in the absence of any commercial or financial relationships that could be construed as a potential conflict of interest.

## Publisher’s Note

All claims expressed in this article are solely those of the authors and do not necessarily represent those of their affiliated organizations, or those of the publisher, the editors and the reviewers. Any product that may be evaluated in this article, or claim that may be made by its manufacturer, is not guaranteed or endorsed by the publisher.

## Supplementary Material

Supplementary Figure 1 Forest plot of preoperative hormone levels from the four medical centers. Horizontal lines indicate the 95% confidence interval. Open black diamonds describe the point estimate (vertical dotted line) and the 95% confidence interval (width of the diamond) for the pooled estimates for all studies.

Supplementary Figure 2 Forest plot of postoperative hormone levels from the four medical centers.

Supplementary Figure 3 Confusion matrixes for predicting deficits of TSH **(A)**, FSH **(B)**, LH **(C)**, PRL **(D)**, and ACTH **(E)** in the optimal models.
