# Supplementary figures and images for: Machine-Learning Prediction of Postoperative Pituitary Hormonal Outcomes in Nonfunctioning Pituitary Adenomas: A Multicenter Study
Source: Front Endocrinol (Lausanne). 2021 Oct 7;12:748725. doi: 10.3389/fendo.2021.748725 (PMC8529112; doi:10.3389/fendo.2021.748725)

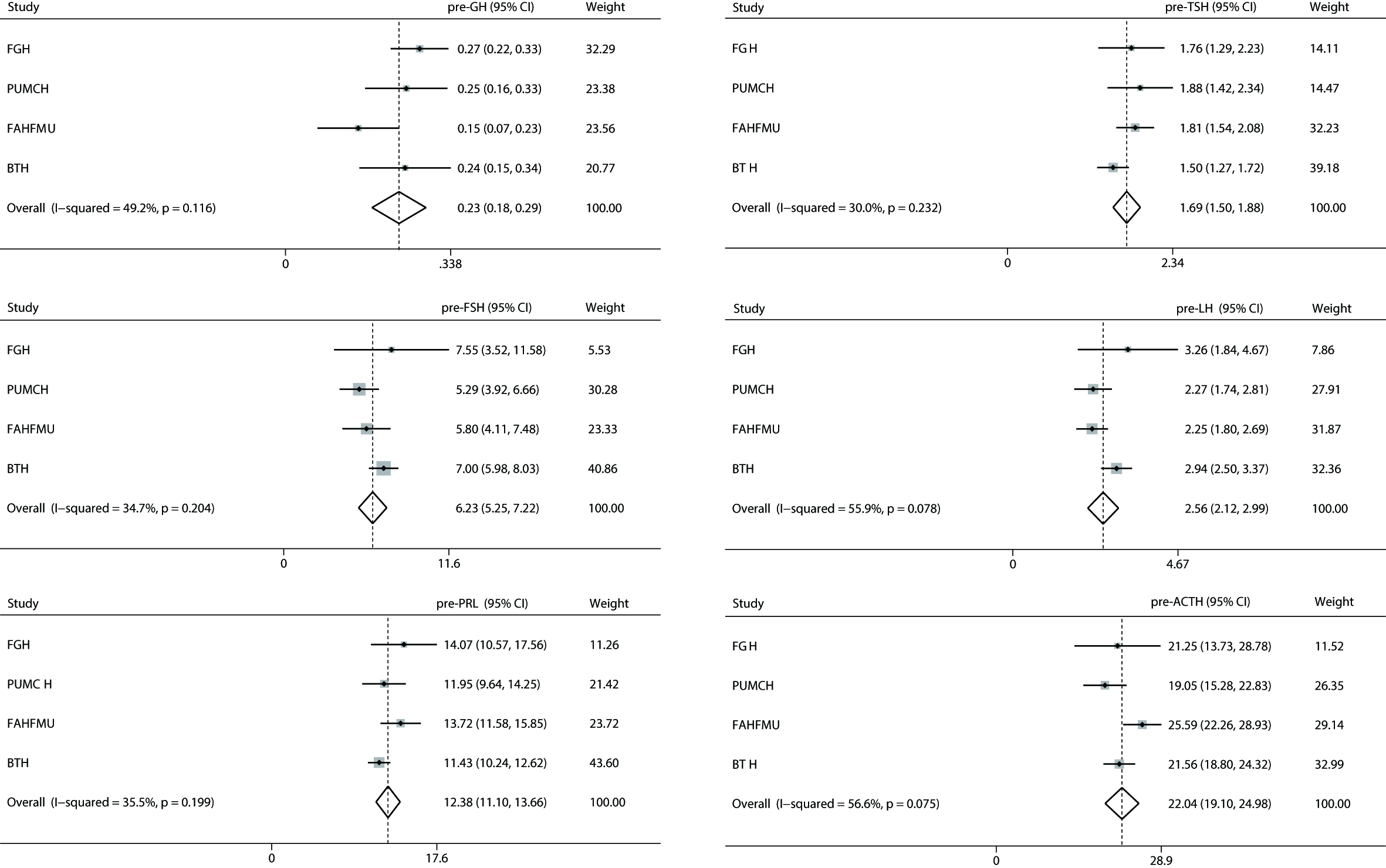

Supplement: Supplementary Figure 1 — Forest plot of preoperative hormone levels from the four medical centers. Horizontal lines indicate the 95% confidence interval. Open black diamonds describe the point estimate (vertical dotted line) and the 95% confidence interval (width of the diamond) for the pooled estimates for all studies. [file Image_1.tif]

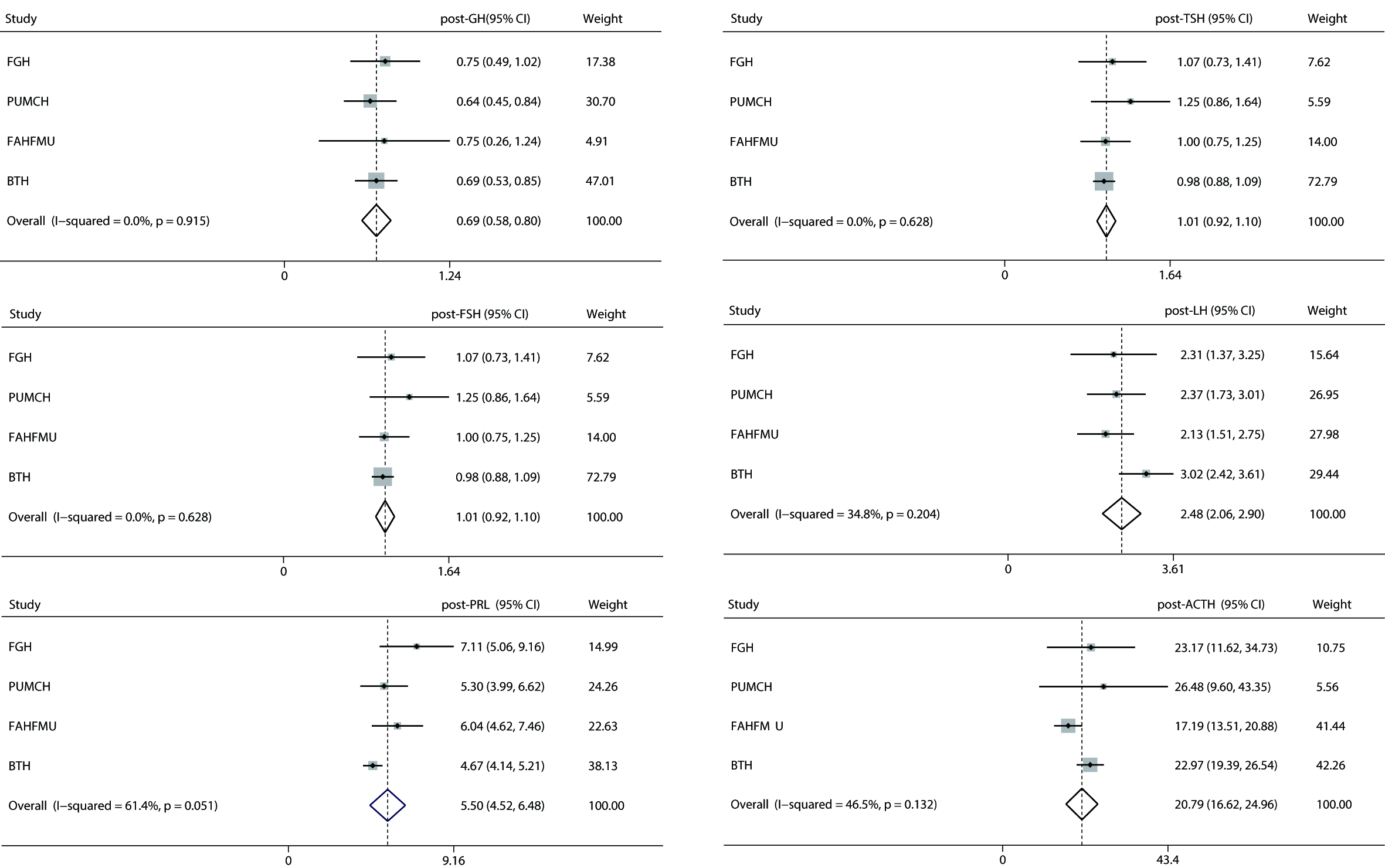

Supplement: Supplementary Figure 2 — Forest plot of postoperative hormone levels from the four medical centers. [file Image_2.tif]

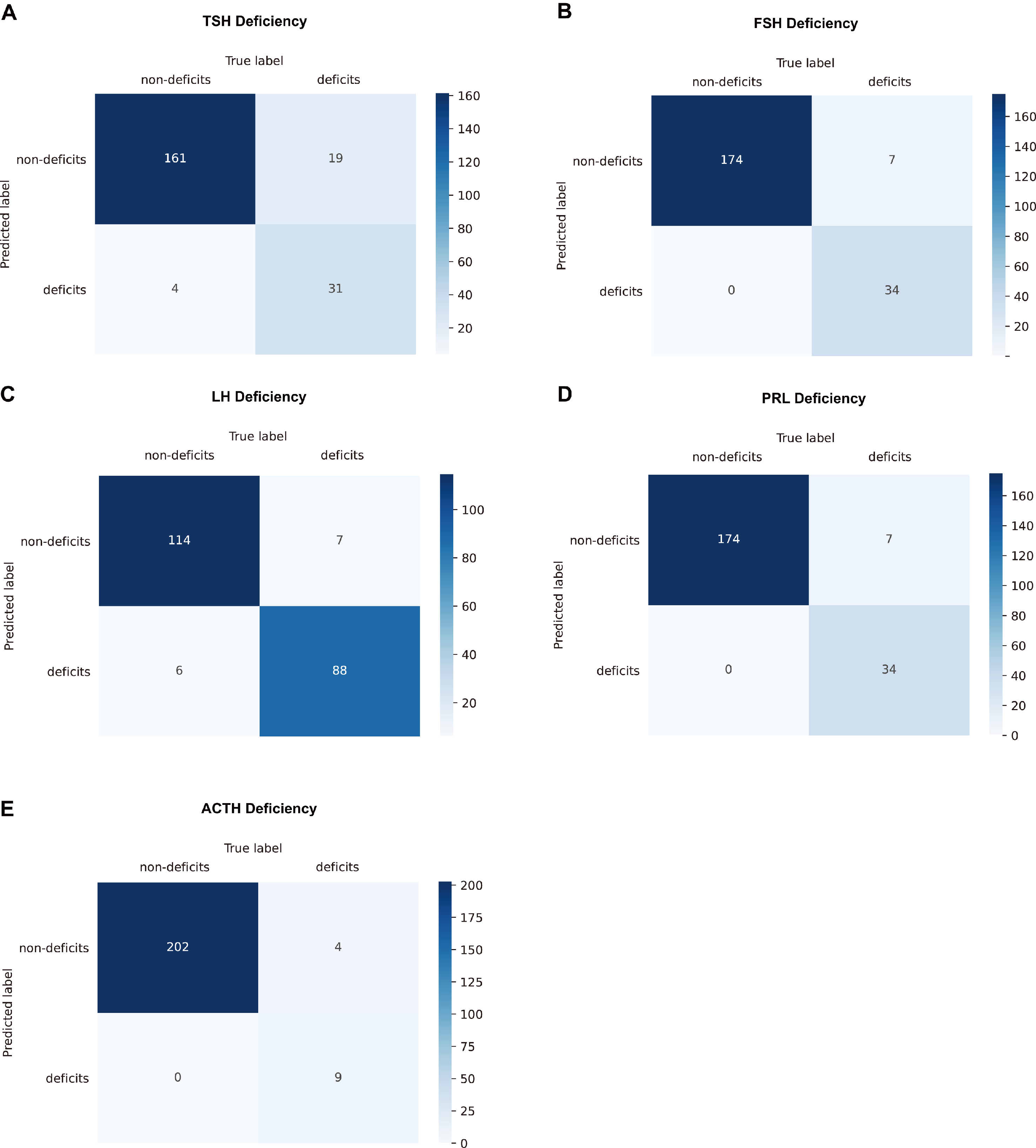

Supplement: Supplementary Figure 3 — Confusion matrixes for predicting deficits of TSH (A), FSH (B), LH (C), PRL (D), and ACTH (E) in the optimal models. [file Image_3.tif]
